# Treatment response and predictors in patients with newly diagnosed epilepsy in Ethiopia: a retrospective cohort study

**DOI:** 10.1038/s41598-019-52574-y

**Published:** 2019-11-07

**Authors:** Kidu Gidey, Legese Chelkeba, Tadesse Dukessa Gemechu, Fekede Bekele Daba

**Affiliations:** 1 Department of Clinical Pharmacy, School of Pharmacy, College of Health Sciences, Mekelle University, Mekelle, Ethiopia; 2 Department of Clinical Pharmacy, School of Pharmacy, Institute of Health, Jimma University, Jimma, Ethiopia; 3 Department of Internal Medicine, School of Medicine, Institute of Health, Jimma University, Jimma, Ethiopia

**Keywords:** Outcomes research, Epilepsy

## Abstract

Epilepsy is a chronic neurological disease with a variable therapeutic response. To design effective treatment strategies for epilepsy, it is important to understand treatment responses and predictive factors. However, limited data are available in Africa, including Ethiopia. The aim of this study was therefore to assess treatment response and identify prognostic predictors among patients with epilepsy at Jimma university medical center, Ethiopia. We conducted a retrospective cohort study of 404 newly diagnosed adult epilepsy patients receiving antiepileptic treatment between May 2010 and May 2015. Demographic, clinical, and outcome data were collected for all patients with a minimum follow-up of two years. Cox proportional hazards model was used to identify predictors of poor seizure remission. Overall, 261 (64.6%) of the patients achieved seizure remission for at least one year. High number of pre-treatment seizures (adjusted hazard ratios (AHR) = 0.64, 95% CI: 0.49–0.83) and poor adherence (AHR = 0.57, 95% CI: 0.44–0.75) were significant predictors of poor seizure remission. In conclusion, our study showed that only about two-thirds of patients had achieved seizure remission. The high number of pre-treatment seizures and non-adherence to antiepileptic medications were predictors of poor seizure remission. Patients with these characteristics should be given special attention.

## Introduction

Epilepsy is one of the common neurological disorders of the brain. It affects more than 69 million people worldwide and about 80% of people with epilepsy are from low- and middle-income countries^1,2^. Epilepsy poses a substantial physical, social, and economic burden for health systems and individuals^3,4^. The risk of premature death due to epilepsy increases two to three times compared to the general population^5^. Mortality could even increase 4–7 times more in people with uncontrolled seizures and its effect is worse in developing countries^6,7^.

Antiepileptic drugs (AEDs) are the mainstay of epilepsy treatment and approximately 70% of patients with newly diagnosed epilepsy achieve seizure remission^8,9^. Although different definitions are given according to different literatures, the most commonly used definition of seizure remission is given by the International League Against Epilepsy, defined as being free from all types of seizures for at least one year^8^. The patient’s response requires careful diagnosis of the types of seizures, knowledge of pharmacotherapy, and patient compliance with treatment^10^. Although responses to the treatment of epilepsy are well studied in developed countries, limited data are available in developing countries, including Ethiopia. The effectiveness and tolerability of AEDs may vary among patients with different ethnicities^11,12^. This makes it difficult to extrapolate treatment response data from developed countries. Besides, the predictive factors of the response to epilepsy treatment are very variable in different populations^13^. Therefore, knowledge of the response to epilepsy treatment and its predictors based on regional data is crucial for developing effective treatment strategies.

Currently, a large number of people with epilepsy live in Ethiopia. It was reported that the prevalence of epilepsy in Ethiopia was 5.2 to 29.5 per 1000 populations^14,15^, but data on epilepsy treatment response and predictors are scarce in this population. The aim of this study was therefore to assess the response to epilepsy treatment and to identify the factors contributing to a poor seizure remission.

## Methods

### Study design and setting

A retrospective cohort study was conducted in patients with newly diagnosed epilepsy on follow-up at the epilepsy clinic of Jimma university medical center. Jimma university medical center is a specialized university hospital in southwest Ethiopia serving for about 15 million people living in the catchment area.

### Study populations

All consecutive adult patients with newly diagnosed epilepsy who have been on regular follow up at the epilepsy clinic of Jimma university medical center from May 2010- May 2015 were recruited and followed until May 2017. The inclusion criteria were all newly diagnosed epilepsy patients, whose age was ≥18 years and patients who were treated for at least two years. Patients were excluded if they had a follow up period less than two years, transferred in from another facility like the health centers or private clinics, and those patients with incomplete medical records (i.e. patients with missing follow up data such as loss of initial chart or patient lost to follow up). Jimma University Medical Center introduced an electronic medical record of patients with epilepsy in 2010. Since then, 639 adult patients with newly diagnosed epilepsy have been registered. Of these, a total of 404 patients met the inclusion criteria and were included in the study.

### Ethical approval

This study was approved by the Institutional Review Board (IRB) of the Jimma University, Institute of Health (reference number: IHRPGC/209/207) and the committee waived the need for informed consent as it was a retrospective study. The study was conducted in accordance with the Declaration of Helsinki. Written permission was obtained to access the patient’s medical records from the hospital’s medical director. The confidentiality of personal information has been strictly preserved. To ensure confidentiality, the name and other identifiers of the patients have not been registered. The data collected was stored in a locked cabinet and only the researchers had access to it.

### Data collection

A data abstraction form has been developed to record relevant information from existing medical records. The data abstraction tool was prepared after reviewing similar studies^13,16,17^. We trained four data collectors on the purpose of the study and data collection methods, including the retrieval of data from patient records. The data collectors then reviewed the records of the eligible patients under the supervision of the research coordinator. Patient demographics (sex, age, family history of epilepsy), disease characteristics (pre-treatment number of seizures, duration of epilepsy, etiology, type of epilepsy), neurological examinations, comorbidities, AED treatments, treatment responses, adherence to treatment, and adverse effects were recorded.

### Treatment

The treatment provided in the current study was performed as part of routine care. Upon diagnosis, an appropriate AED was chosen considering seizure type, side effects, and drug interaction profiles. Monotherapy was the first antiepileptic drug used in all the patients. Initially, the minimum effective dose was prescribed and dose increases were made to control seizures or up to the maximum tolerated dose. The treatment regimens have been modified according to the patient’s response and tolerance. If the patient developed intolerable side effects with the initial drug or if the drug does not improve the seizure outcome, it was replaced with an alternative drug. However, if the initial monotherapy was tolerated and improves the seizure outcome, but did not completely provide seizure remission, a combination of drugs was prescribed. The AEDs prescribed in our study were the old AEDs (phenytoin, phenobarbital, carbamazepine, and valproate). Adherence to AEDs was checked at each visit and recorded. More than 95% of adherence to treatment is needed to prevent epileptic seizures and omitting a single dose can provoke a seizure^18^. Therefore, we classify the patient’s adherence based on the all-or-nothing rule. The patient was considered adherent if there was no missed dose.

### Follow-up

During the first six months after starting treatment, patients were followed up at the epilepsy clinic every 2 to 4 weeks. Thereafter, patients underwent follow-up visits at least every 3 months. As our data were collected during standard medical care, patients were evaluated every 4 weeks for the first 6 months and every 3 months thereafter. At each follow-up visit, the response to AEDs treatment (i.e. the patient/ caregiver was asked to report the number of seizures between visits to the clinic), prescribed medications, adverse events, and adherence were routinely recorded. These data were extracted into a data abstraction sheet developed for this study, and the response to drug therapy was assessed at the end of the follow-up. The follow-up period was calculated from the date of the initial diagnosis to the last visit or death, whichever comes first. The final assessment of seizure control was conducted after at least two years of follow-up. For comparison purposes, we divided the patients into two groups based on achieving remission of seizures over one year. Patients who had seizure remission for at least one year were classified in the “remission” group and the remaining patients in the “non-remission” group.

### Definitions

In this study, we followed the definition of epilepsy published in 2014 by the International League for Epilepsy (ILAE) ILAE^19^. Accordingly, epilepsy was defined by (1) at least two unprovoked seizures occurring >24 hours apart; (2) an unprovoked seizure and a likelihood of further seizures similar to the general recurrence risk after two unprovoked seizures occurring in the next 10 years; and (3) diagnosis of the epilepsy syndrome. Investigations were performed as clinically indicated, including electroencephalography and brain imaging (computed tomography and/or magnetic resonance imaging) to assist in the diagnosis, classification of epilepsy and screening for structural abnormalities. The types of epilepsy have been classified into generalized, focal and unclassified. The etiology of epilepsy was classified as genetic, structural/metabolic, unknown, according to the ILAE classification criteria for epilepsy and epileptic syndromes^20^.

Seizure remission was defined as an attainment of at least one-year seizure freedom. Early remission was defined as an achievement of a one-year seizure-free period started immediately or in the first 6 months. Late remission was defined as achievement of a one-year seizure-free period, starting later than 6 months after treatment initiation. After achievement of the one-year seizure-free period, a patient may become seizure free and persisting until the end follow-up visit (Sustained remission) or recurrence of seizures (relapse) may occur. Remitting-relapsing course was defined as a seizure fluctuating between periods of seizure freedom and relapse. Remission at last visit was defined as at least a 1-year seizure free period at the last follow-up visit, with or without previous relapses. A worsening course was defined as seizure continues after relapsing. No remission was defined as no seizure remission through the entire follow-up period. According to these definitions, outcome patterns of seizure remissions and relapses were reported in four main categories: Early and sustained remission, late but sustained remission, remitting-relapsing course (the patients had early or late remission but relapse occurred) and no remission.

### Statistical analysis

Data were entered into EPI-Data version 4.2.0.0 and exported to SPSS (IBM SPSS Statistics for Windows, Version 21.0. Armonk, NY: IBM Corp) for analysis. We presented the data using the mean (SD) and median for continuous variables or frequency for categorical variables. A univariable Cox proportional hazards model was performed to determine the association of each independent variable with seizure remission. Subsequently, variables with a p-value < 0.25 in the univariable analysis were included in the multivariable Cox proportional hazards model to identify independent predictors of remission. A p-value of < 0.05 (α = 0.05) was used to declare a statistically significant association.

## Results

### Demographic and clinical characteristics of patients

**Figure 1:**
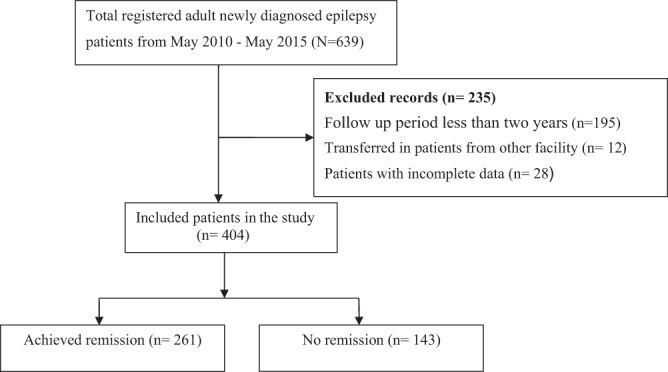
Flow diagram of patients included in the study.

We identified 639 patients with newly diagnosed epilepsy during the study period. Of these, 404 patients met our inclusion criteria and were included in the final analysis (Fig. 1).

Of the patients included, 60.1% were male. The mean age at onset of seizure was 27.4 years (Standard deviation (SD) = 11.2). A family history of epilepsy was present in 9.9% of cases. Generalized epilepsy was the predominant type of epilepsy, accounting for 78.7% of cases. More than half (63.86%) of the patients had more than five episodes of seizures at the time of diagnosis. The cause of epilepsy was genetic in 9.9% of patients, structural/metabolic in 32.7% of patients and unknown in 57.4% of patients. Comorbid disease (including depression, anxiety, bipolar disorder, psychosis, human immunodeficiency virus, dyspepsia, asthma) was present in 13.4% of patients. Status epilepticus occurred in 4.7% of patients. More than sixty percent of the patients adhered to the treatment of epilepsy. The median duration of the follow-up was 44.0 months (3.6 years) (range 2–7 years) (Table 1).

**Table 1:** Demographic and clinical characteristics of patients.

Characteristics	Category	Remission (%)	No remission (%)	Total n (%)
Sex	Male	161(39.9)	82(20.3)	243(60.1)
	Female	100(24.8)	61(15.1)	161 (39.9)
Family history of epilepsy	Yes	23(5.7)	17(4.2)	40(9.9)
	No	158(39.1)	75(18.6)	233 (57.7)
	NA	80(19.8)	51(12.6)	132 (32.4)
Age at onset of seizure	<30	168(41.6)	97(24)	265 (65.6)
	30–44	59(14.6)	34(8.4)	93 (23)
	≥45	34(8.4)	12(3)	46 (11.4)
Pre-treatment number of seizures	< = 5 seizures	115(28.5)	31(7.7)	146(36.1)
	>5 seizures	146(36.1)	112(27.7)	258(63.9)
Pre-treatment duration	≤12 months	165(40.8)	77(19.1)	242 (59.9)
	>12 months	96(23.8)	66(16.3)	162 (40.1)
Epilepsy type	Generalized	205(50.7)	113(28)	318 (78.7)
	Focal	14(3.5)	6(1.5)	20 (5.0)
	Unclassified	42(10.4)	24(5.9)	66 (16.3)
Etiology	Genetic	23(5.7)	17(4.2)	40 (9.9)
	Structural/metabolic	93(23.0)	39(9.7)	132 (32.7)
	unknown cause	145 (35.9)	87(21.5)	232(57.4))
Neurologic examination	Normal	234(57.9)	118(29.2)	352 (87.1)
	Abnormal	27(6.7)	25(6.2)	52 (12.9)
Adherence to AEDs	Good	179(44.3)	67(16.6)	246 (60.9)
	Poor	82(20.3)	76(18.8)	158 (39.1)
Adverse events	Yes	93(23)	70(17.3)	163 (40.3)
	No	168(41.6)	73(18.1)	241 (59.7)
Comorbidities	Yes	38(9.4)	16(4)	54 (13.4)
	No	223(55.2)	127(31.4)	350(86.6)
Duration of the follow-up	Median in months (IQR)	44(28, 67.75)		
	Range in months	24–84		

AEDs: Anti-epileptic drugs, IQR: interquartile range.

### Prognostic patterns of patients with newly diagnosed epilepsy

Overall, 261 (64.6%) of the patients achieved remission of seizures for at least one year.

The remaining 143 patients (35.4%) never experienced remission while continuing treatment.

**Figure 2:**
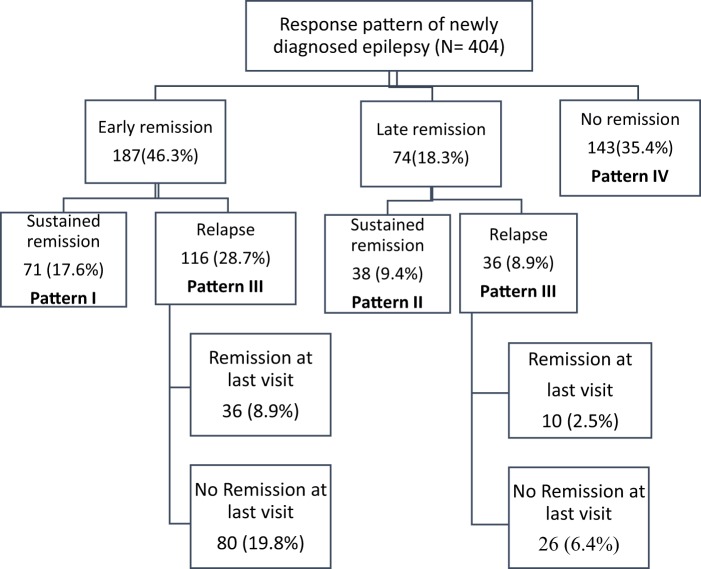
Prognostic patterns of patients with newly diagnosed epilepsy.

As shown in Fig. 2 an analysis was made to look for the epilepsy prognostic patterns. These prognostic patterns were categorized into four different patterns: Early and sustained remission (pattern I), late but sustained remission (pattern II), remitting-relapsing course (pattern III) and no remission (pattern IV). Early and sustained remission was observed in 17.6% and late but sustained remission in 9.4% of patients. Remitting-relapsing course was observed in 37.6% of patients. A total of 35.4% of patients never achieved seizure remission for one full year during follow-up and were categorised in the no-remission pattern (Fig. 2).

### Analysis of predictors of poor seizure remission

A univariable Cox proportional hazards analysis was performed to determine the association of independent variables with the seizure remission and demonstrated that the number of seizures prior to AED treatment (crude hazard ratios CHR) = 0.57, 95% CI: 0.44–0.72), adherence to AEDs (CHR = 0.53, 95% CI: 0.41–0.69), and the presence of adverse event (CHR = 0.67, 95% CI: 0.52–0.86) were significantly associated with poor seizure remission (Supplementary Table S1). However, in the multivariable Cox proportional hazards model, only the number of pretreatment seizures and poor adherence to AED therapy were associated with poor seizure remission. Patients with a higher pretreatment seizures had a 36% reduced chance of achieving seizure remission (AHR = 0.64, 95% CI: 0.49–0.83), and patients with a poor treatment adherence were 43% less likely to achieve seizure remission (adjusted hazard ratios (AHR) = 0.57, 95% CI: 0.44–0.75) (Table 2).

**Table 2:** A multivariable Cox proportional hazards analysis of predictors of poor seizure remission among patients with epilepsy.

Characteristics	Category	CHR (95% CI)	P-value	AHR (95% CI)	P-value
Family history of epilepsy	No	1			
	Yes	0.71 [0.46, 1.10]	0.12	0.88 [0.56, 1.37]	0.56
	NA	0.79 [0.61, 1.04]	0.09	0.81 [0.62, 1.06]	0.13
Age category at onset of seizure	<30	1			
	30–45	1.03 [0.76, 1.38]	0.87	1.00 [0.74, 1.35]	0.99
	≥45	1.31 [0.90, 1.89]	0.15	1.26[0.87, 1.84]	0.23
Pretreatment number of seizures	≤5	1			
	>5	0.57 [0.44, 0.72]	<0.001	**0**.**64 [0**.**49**, **0**.**83]**	0.001
Neurologic examination	Normal	1			
	Abnormal	0.71[0.48, 1.06]	0.09	0.74 [0.49, 1.12]	0.15
Adherence	Good	1			
	Poor	0.53 [0.41, 0.69]	0.001	**0**.**57 [0**.**44**, **0**.**75]**	<0.001
Adverse events	Yes	0.67 [0.52, 0.86]	0.02	0.78 [0.59, 1.02]	0.74
	No	1			
Comorbidities	Yes	1.24[0.88, 1.76]	0.21	01.31 [0.91, 1.89]	0.15
	No	1			

## Discussion

To our knowledge, this is the largest study conducted in Ethiopia on a cohort of patients of reasonable sample size and over a longer follow-up period, dealing with epilepsy response and predictors of poor seizure remission. Generalized epilepsy was the predominant type of epilepsy, accounting for 78.7% of cases. This is inconsistent with most epidemiological studies conducted in China^21^, Egypt (47.6%)^16^, Italy (34.8%)^22^ and Istanbul (47.0%)^23^. Similar high rates were observed, however, in Ethiopia (Amhara region) (72.91%)^24^, Ethiopia (Tigray region) (84.4%)^25^, Senegal (78%)^26^, and South Africa (96%)^27^. Factors including the etiological differences and ethnic difference might contribute for this discrepancy. Furthermore, the reason for this high proportion of generalized epilepsy in developing countries, particularly in Africa, might be influenced by the poor availability of medically sophisticated diagnostic tests, the level of training of the neurologists in the interpretation of EEG and, therefore, misclassification could also be expected^28^.

We observed that only 261 patients (64.6%) had seizure remission, similar to the rate observed in a large cohort of Scottish patients^29^ (63.7%) and suggest that a significant proportion of patients had not achieved remission. However, this result was lower than that of a study conducted in China (80%)^21^. Similarly, a slightly higher remission rate (69.1%) was reported by Shen *et al*.^9^. In contrast to these findings, only 45% of patients living in rural areas of China had a remission of seizures for at least one year^17^. A possible explanation for the discrepancy of these studies could be differences in methodology, such as the definitions used for remission and duration of follow-up, type of medications used, exclusion of non-adherent patients in some studies and the possibility of genetic influence.

According to seizure remission patterns, 46% of our patients entered early remission and about 18% entered late remission. This result is consistent with the results of Shen *et al*., who reported early remission in 51.6% of patients and delayed remission in 17.5% of patients^9^. Another study in Italy also found that 56.2% of patients had early remission^30^. More than one-third of our patients never went into remission during the follow-up period. Similarly, a significant proportion of patients had not experienced remission in earlier studies conducted in China (31%)^9^ and Scotland (25%)^13^. We found that remitting-relapsing course was the most common (37.6%) prognostic pattern in line with an Italian study^31^. This indicates that achieving a seizure remission at any time after the start of treatment does not exclude further relapses and adequate follow-up is important for all patients, including patients in remission.

Another aim of the study was to identify the predictor of poor seizure remission in patients with epilepsy. We demonstrated that patients with a high number of pre-treatment seizures were associated with poor treatment seizure remission. In line with current findings, previous studies have shown that a higher number of seizures prior to treatment was associated with lower chances of remission^30–35^. The role of the number of seizures prior to treatment is based on the theory that prolonged and recurrent seizures may contribute to neuronal damage and the development of new epileptic foci^36^. In addition, a high number of pre-treatment seizures may correlate with the intrinsic severity of the disease which is more likely to give a poor response to drug therapy^37^. This indicates that a seizure must be recognized soon and appropriate treatment is required to prevent injury, premature death, cognitive impairment, and psychosocial stigma^34,35^. The patient’s poor adherence to antiepileptic therapy was also a significant predictor of poor remission. This finding is consistent with other similar studies and suggests that lack of adherence is an important cause of uncontrolled seizures^38,39^.

The findings of our study have some important implications for clinical practice. Our study found that a high number of pre-treatment seizures was a predictor of poor seizure remission. The high number of seizures before treatment could be due to different factors related to the patient.

According to an earlier study in Ethiopia, the initial choice of the place of epilepsy treatment for the majority (60%) of the patients was traditional and religious healers rather than modern therapies^40^. Health care-seeking behavior was also poor and the majority (87%) of patients had not been treated with AEDs in a community-based study^15^. Another study on stigma related to epilepsy revealed that the majority of participants did not want to disclose their disease to the community^41^. All these factors could contribute to poor treatment seeking behaviors until several seizures occur. In the current study, we found that a high number of pretreatment seizures were significantly associated with poor remission. However, the pretreatment duration of epilepsy was not significantly associated with poor seizure remission. This suggests a high number of pretreatment seizure in a short or long period of time is the most important factor rather than the longer pre-treatment duration of seizures. Therefore, activities such as awareness-raising campaigns and the dissemination of information through the media could help patients seek treatment of epilepsy before subsequent several seizures occur. Non-adherent patients also had a high risk of poor remission. Therefore, all health care providers must provide health education to prevent nonadherence.

Finally, some important limitations must be taken into account in our study. Due to the retrospective nature of the study, the data extracted from the secondary source were prone to the absence of important characteristics such as family history. However, attempts have been made to exhaustively search for variables throughout the record, from the initial visit to the end of the follow-up. The other limitation of this study was the inclusion of non-adherent patients that could reduce the remission rate. However, excluding these patients may result in an unpowered sample, since a large number of patients were not adherent. In addition, the inclusion of these patients will help to mimic the real-world experiences of our patients. Due to these limitations, our results should be interpreted with caution and further prospective studies are suggested.

## Conclusion

Our study showed that only about two-thirds of patients had achieved seizure remission during follow-up. In addition, patients in remission had a high risk of relapse, which made the remitting-relapsing course the most common outcome pattern. The high number of pre-treatment seizures and nonadherence with AEDs were independent predictors of poor remission of seizures. Patients with these characteristics should be given special attention.

## Supplementary information

Supplementary Table S1

## Data Availability

The dataset analyzed during the current study is available from the corresponding author upon reasonable request.
