# Author Correction: Treatment response and predictors in patients with newly diagnosed epilepsy in Ethiopia: a retrospective cohort study

**DOI:** 10.1038/s41598-020-59359-8

**Published:** 2020-02-07

**Authors:** Kidu Gidey, Legese Chelkeba, Tadesse Dukessa Gemechu, Fekede Bekele Daba

**Affiliations:** 1 Department of Clinical Pharmacy, School of Pharmacy, College of Health Sciences, Mekelle University, Mekelle, Ethiopia; 2 Department of Clinical Pharmacy, School of Pharmacy, Institute of Health, Jimma University, Jimma, Ethiopia; 3 Department of Internal Medicine, School of Medicine, Institute of Health, Jimma University, Jimma, Ethiopia

Correction to: *Scientific Reports* 10.1038/s41598-019-52574-y, published online 07 November 2019

The original version of this Article contained a typographical error in the spelling of the author Legese Chelkeba, which was incorrectly given as Legesse Chelkeba. This has now been corrected in the PDF and HTML versions of the Article, and in the accompanying Supplementary Information file.

