# Supplementary material for: Treatment response and predictors in patients with newly diagnosed epilepsy in Ethiopia: a retrospective cohort study
Source: Sci Rep. 2019 Nov 7;9:16254. doi: 10.1038/s41598-019-52574-y (PMC6838070; doi:10.1038/s41598-019-52574-y)
Supplement: Supplementary file 1 — Supplementary Table S1 [file 41598_2019_52574_MOESM1_ESM.docx]

# Treatment response and predictors in patients with newly diagnosed epilepsy in Ethiopia: a retrospective cohort study

Kidu Gidey^1*^, Legese Chelkeba^2^, Tadesse Gemechu^3^, Fekede Bekele^2^

^1^Department of Clinical Pharmacy, School of Pharmacy, College of Health Sciences, Mekelle University, Mekelle, Ethiopia.

^2^Department of Clinical Pharmacy, School of Pharmacy, Institute of Health, Jimma University, Jimma, Ethiopia.

^3^Department of Internal Medicine, School of Medicine, Institute of Health, Jimma University, Jimma, Ethiopia.

^*^Corresponding author: E-mail: [kidu.gidey@mu.edu.et](mailto:kidu.gidey@mu.edu.et) (KG)

Supplementary table S1: A univariate Cox proportional hazards analysis of association of each independent variable with poor seizure remission among patients with epilepsy

| Characteristics | Category | CHR (95% CI) | P value |
| --- | --- | --- | --- |
| Sex | Female | 1 |  |
|  | Male | 1.11 [0.86, 1.42] | 0.42 |
| Family history of epilepsy | No | 1 |  |
|  | Yes | 0.71 [0.46, 1.10] | 0.12 |
|  | NA | 0.79 [0.61, 1.04] | 0.09 |
| Age category at onset of seizure | <30 | 1 |  |
|  | 30-45 | 1.03 [0.76, 1.38] | 0.87 |
|  | ≥ 45 | 1.31 [0.90, 1.89] | 0.15 |
| Pretreatment number of seizures | ≤ 5 | 1 |  |
|  | >5 | 0.57 [0.44, 0.72] | 0.000 |
| Pre-treatment duration | ≤ 12 months | 1 |  |
|  | >12 months | 0.88 [0.68, 1.13] | 0.32 |
| Seizure type | Generalized | 1 |  |
|  | Focal seizure | 1.27 [0.74, 2.19] | 0.39 |
|  | Undetermined | 1.03 [0.74, 1.43] | 0.88 |
| Etiology | Genetic | 1 |  |
|  | Structural/metabolic | 1.18[0.69, 1.99] | 0.54 |
|  | Unknown | 0.80[0.46, 1.40] | 0.44 |
| Neurologic examination | Normal | 1 |  |
|  | Abnormal | 0.71[0.48, 1.06] | 0.09 |
| Adherence | Good | 1 |  |
|  | Poor | 0.53 [0.41, 0.69] | 0.001 |
| Adverse events | Yes | 0.67 [0.52, 0.86] | 0.02 |
|  | No | 1 |  |
| Comorbidities | Yes | 1.24[0.88, 1.76] | 0.21 |
|  | No | 1 |  |
| Status epilepticus | Yes | 1.04 [0.58, 1.85] | 0.90 |
|  | No | 1 |  |
